# Demand Side Factors Associated With Quality Antenatal Care Services: A Case Study of Lusaka District, Zambia

**DOI:** 10.3389/fpubh.2018.00285

**Published:** 2018-10-09

**Authors:** Brave M. Katemba, Phoebe Bwembya, Twaambo E. Hamoonga, Mumbi Chola, Choolwe Jacobs

**Affiliations:** ^1^Department of Community and Family Medicine, School of Public Health, University of Zambia, Lusaka, Zambia; ^2^Department of Epidemiology & Biostatistics, School of Public Health, University of Zambia, Lusaka, Zambia

**Keywords:** antenatal care, maternal mortality, quality of antenatal care, infant mortality, high quality antenatal care

## Abstract

**Background:** The quality of antenatal care (ANC) a woman receives during pregnancy is crucial to both the child and the mother's life. It has been established that providing high-quality ANC can save lives and has a positive impact on postnatal health care services. However, the quality of ANC in Zambia requires attention as maternal and neonatal mortality rates are still unacceptably high with Lusaka district not being left out of the problem.

**Methods:** Using a cross-sectional study design, the main aim of this study was to determine the demand side factors associated with high-quality antenatal care among pregnant women in Lusaka. It also estimated the proportion of women who received high-quality ANC during their last antenatal visit. Multifactorial logistic regression model was fitted in STATA version 13 to predict the demographic, socio and economic factors that influence the quality of ANC.

**Results:** It was established that only 47.1% of pregnant women received high-quality ANC while 52.9% received low quality. Six key ANC interventions were considered, among which urine (36.7%) and blood (46.8%) testing were the least received basic components of ANC. After adjusting for the effect of other factors, women with secondary education had higher odds of receiving high-quality ANC than women with primary level of education (OR = 1.98; 95% CI: 1.24–3.14). Women staying with their husband/partners had lesser odds of receiving high quality ANC compared to those that were not staying with their partners (OR = 0.47; 95% CI: 0.28–0.79).

**Conclusion:** The quality of antenatal care received by pregnant women in Lusaka is low. Continued efforts to improve the delivery of basic ANC services such as blood and urine testing is required to improve the quality of healthcare services provided by medical personnel at all levels.

## Introduction

The quality of antenatal care (ANC) that pregnant women receive in Zambia continues to be poor despite several interventions. Research has shown that only 29% of women in Zambia receive high-quality antenatal care (1). Low ANC quality has serious adverse effects on the health of pregnant women and contributes to the high number of maternal deaths (2). Currently, Zambia remains one of the countries with unacceptably high Maternal Mortality rates with 323–474 maternal deaths per 100,000 live births in 2014 (3). The maternal mortality rate for Lusaka province has been increasing since 2013 with over 70% of maternal deaths in the province being recorded in Lusaka District.

To improve the quality of ANC, the government, through the Ministry of Health, adopted the Focused Antenatal Care (FANC) model known to improve quality and utilization of ANC services. In 2005, the Zambian National Health Strategic Plan included key components of the new model (4). The expectation is that FANC would go a long way in improving the quality of ANC. In this study, high quality ANC is defined as having received at least five antenatal interventions among the following six: blood testing, urine testing, blood pressure measurements, abdominal examination, weighing measurement, and information on pregnancy related complications (5, 6).

In the assessment of quality of care, Donabedian proposed a framework which distinguishes between demand and supply side attributes (7). Demand side factors focus on factors that influence clients' decision to demand for a particular service. Among the demand side factors that influence quality of ANC services is parity, education level of a pregnant woman, marital status, employment status, birth order, age, time taken to the health facility as well as client's perception of the service provided (8, 9).

In Zambia, very little is known about the factors that affect quality of antenatal care and as such, this study aimed at determining the demand side factors that influence quality of ANC.

## Methods

A cross sectional study was conducted in Lusaka district of Zambia; the district has been described in details elsewhere (10). However, it suffices to state here that the district has a total number of 39 health facilities providing primary health care.

Geographically, the health facilities are clustered into eight zones based on their geographical setting within the district. The study population comprised of all consenting pregnant women aged 15–49 years attending ANC either in the first, second, or third trimester between October and November 2016. All pregnant women admitted at the health facility or referred to a general hospital at the time of the study were excluded from the study.

### Sampling technique and sample size determination

A multi-stage sampling technique was used to select the 480 study participants. In the first stage, simple random sampling method using a lottery approach was used to randomly select eight health facilities, one from each zone. Different health facilities had different average number of women attending ANC per month, as such, data collectors divided the total population of women who reported for ANC in the preceding data collection month by the desired sample size to obtain the interval of women to be selected for exit interviews. In the second stage, systematic sampling was used to select women for exit oral interviews by means of a closed ended questionnaire administered by a data collector. The process of systematic sampling involved the selection of one pregnant women as a fixed starting point and later obtaining subsequent women using a constant interval between the required sample size.

### Sample size calculation

$$\begin{array}{l} \text{n}=\frac{{\text{z}}^{2}p\left(deff\right)\left(1-p\right)}{e} \end{array}$$

*z* = Is standard normal variate at 5% type 1 error (0.05). This was set at 1.96 to correspond to 95% confidence level.
*p* = Is the proportion used in the estimation formula in our case the *p* value used is 0.29 (29%), based on previous studies, the proportion of women who had high quality antenatal care services was estimated to be 29% in Zambia (1).
*e* = Is a measure of precision, thus the margin of error. In this study the margin of error is set at 0.05.
Deff = is the design effect set at 1.5, this was chosen arbitrarily because no literature was found on the similar study (factors associated with quality of ANC) in Zambia.
The estimated sample size was: $\text{n}=\frac{1.96^{2}0.29\left(1.5\right)\left(1-0.29\right)}{0.05^{2}}=475$
Adjusting the sample size upwards for assuming non-response rate (*r*), the sample size was adjusted as follows:
*n*_*f*_=$\frac{n}{r}$
Where *n*_*f*_ is the final sample size and *r* is the response rate in decimals which is 95.8% (0.958) for urban women in the ZDHS of 2013–2014 (3).
$n_{f}=\frac{475}{0.958}\approx 495$(total sample size)

Considering the cost, the margin of error and scientific validity factors, the overall sample size of 495 was large enough to give reliable results.

### Outcome variable

To determine the binary outcome variable, data on the checklist was scored in MS excel to establish whether a pregnant woman received “High” or “Low” quality ANC. A woman was classified as having received high-quality ANC if they received five components of the six, while low quality meant receiving less than five of the mentioned components. The six ANC components from which the outcome variable was generated are: blood testing, urine testing, blood pressure measurements, abdominal examination, weighing measurement and information on pregnancy-related complications.

### Explanatory variables

Explanatory variables considered were marital status, parity, the age of the pregnant women, level of household income, the area of residence, employment status, and time taken to get to the facility.

### Data analysis

Bivariate analysis was used to examine the demographic and socio-economic factors that were associated with quality of ANC pregnant woman received. All variables that were significant (*p* < 0.05) at bivariate level were then retained in the multifactorial logistic regression model. The research further used a backward elimination method by removing all variables with the least significant global *p*-value. This process was repeated until the model only had variables with a *p*-value of < 0.05. Data analysis of this study was done in STATA 13.1.

### Ethics

Ethical clearance was obtained from the University of Zambia Biomedical Research Ethics Committee (Ref: 037-06-16). Written permission was also requested from Lusaka District Medical Office and the Ministry of Health before data collection began. All participants were informed about the purpose of the study and completed a consent form before participating. The participants also had the right to decline from participating and withdrawing from the study at any point.

## Results

The estimated sample size for this study was 495 pregnant women attending ANC. However, only 480 women completed the survey indicating a response rate of 97%.

Table 1 presents the background characteristics of the 480 women who completed the exit interviews. About one third (30.8%) of the respondents were aged between 20 and 24 years while only 3.5% women were aged above 40 years. The average age of the study population was 26 years while the average number of children a woman had was one child (SD 1.5). Over two thirds (68.1%) of the women were married and residing in high densely populated areas (68.9%). The study also showed that 8 in 10 (84.7%) women interviewed had attended school at some point in their lifetime although slightly above half (51.5%) only went as far as primary level. About 59.2% women were informally employed with K1000–K3000 being the average household income. Over one third (37.7%) of the women had no children but pregnant for the first time.

**Table 1:** Background characteristics of respondents.

**Variables**	**Respondents *(n)***	**Percentage (%)**
**AGE GROUP**		
Below 20	84	18.3
20–24	141	30.8
25–29	94	20.5
30–34	90	19.7
35–39	33	7.2
40+	16	3.5
Mean age	25.6 years	(SD 1.5)
**MARITAL STATUS**		
Not married	151	31.9
Married	323	68.1
**AREA OF RESIDENCE**		
Low density	28	5.9
Medium density	120	25.2
High density	328	68.9
**EMPLOYMENT STATUS**		
Formal	82	40.8
Informal	119	59.2
**EVER ATTENDED SCHOOL**		
Yes	305	84.7
No	55	15.3
**HIGHEST LEVEL OF SCHOOL ATTENDED**		
Primary	189	51.5
Secondary	133	36.4
Tertiary	45	12.3
**NUMBER OF CHILDREN**		
None (first pregnancy)	177	37.7
1	112	23.9
2	82	17.5
3+	98	21
Mean number of children	1.4	
**HOUSEHOLD LEVEL OF INCOME**		
Less than K500	34	10.2
K500–K900	52	15.6
K1000–K1900	91	27.3
K2000–K3000	91	27.3
Above K3000	66	19.8

*SD, standard deviation; Household level of income (US dollar 1 = K10.00)*.

### Antenatal care services

**Figure 1:**
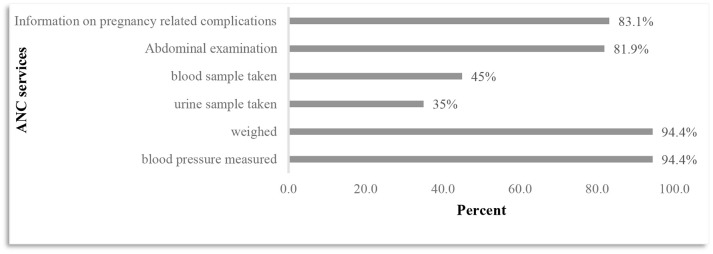
Antenatal care services received by pregnant women.

Figure 1 shows the six key ANC interventions used to determine the quality of ANC. It can be shown that the widely provided services was blood pressure measurements and weighing with 94.4% pregnant women receiving both services. Testing services were the least provided interventions as only 45 and 35% pregnant women received the mentioned tests, respectively.

### Bivariate logistic regression analysis

Table 2 shows the bivariate logistic regression analysis on factors associated with quality ANC received by pregnant women during their last ANC visit. Factor variables included all demographic and socio-economic factors.

**Table 2:** Unadjusted (Bivariate logistic analysis) factors associated with high quality of antenatal care.

**Characteristics/factors**	**Study sample (%)**	**Odds ratio (OR) (95% CI)**	***P*-value**
**AGE**			
Below 20	18.3	1.0	
20–24	30.8	0.57 (0.32–1.01)	0.058
25–29	20.5	0.38 (0.19–0.75)	0.006^*^
30–34	19.7	0.38 (0.19–0.74)	0.004^*^
35–39	7.2	0.27 (0.11–0.68)	0.006^*^
40+	3.5	3.03 (0.62–14.9)	0.079
**MARITAL STATUS**			
Not married	31.9	1.0	
Married	68.1	0.52 (0.34–0.80)	0.003^*^
**AREA OF RESIDENCE**			
High density	68.9	1.0	
Medium density	25.2	1.37 (0.84–2.22)	0.205
Low density	5.9	0.59 (0.20–1.76)	0.347
**EMPLOYMENT STATUS**			
Formal	40.8	1.0	
Informal	59.2	1.12 (0.50–2.47)	0.781
**EVER ATTENDED SCHOOL**			
No	15.3	1.0	
Yes	84.7	0.62 (0.35–1.10)	0.103
**HIGHEST LEVEL OF SCHOOL ATTENDED**			
Primary	51.5	1.0	
Tertiary	12.3	1.42 (0.74–2.74)	0.292
Secondary	36.4	1.97 (1.26–3.10)	0.003^*^
**CURRENTLY LIVING WITH PARTNER**			
No	35.2	1.0	
Yes	64.8	0.47 (0.30–0.74)	0.001^*^
**NUMBER OF CHILDREN**			
0 (none)	42	1.0	
1	22.8	0.56 (0.32–0.96)	0.034^*^
2	11.8	0.37 (0.18–0.75)	0.006^*^
3+	23.4	0.56 (0.34–0.96)	0.036^*^
**HOUSEHOLD LEVEL OF INCOME**			
Less than K500	10.2	1.0	
K500–K900	15.6	3.06 (1.13–8.28)	0.028^*^
K1000–K1900	27.3	2.19 (0.86–5.59)	0.099
K2000–K3000	27.3	3.95 (1.56–9.97)	0.004^*^
Above K3000	19.8	4.35 (1.66–11.39)	0.003^*^

* *p < 0.05; Household level of income (US dollar 1 = K10.00)*.

Results in Table 2 show that women in the age groups between 25 and 39 years had lower odds of receiving high quality ANC compared to women aged below 20 years.

Married women were 48% less likely to receive high-quality ANC compared to women who were not married (OR = 0.52; 95% CI: 0.34–0.80). With regard to education, women with secondary school education were two times more likely to receive high-quality ANC than women with primary education (OR = 1.97; 95% CI: 1.26–3.10).

Women who were living with their male partners were less likely to receive high quality ANC compared to those who were not living with their partners (OR = 0.47; 95% CI: 0.30–0.74). High parity reduced the odds of a woman receiving high quality ANC as women with more than three children were almost 50% less likely to receive high quality ANC than women who were pregnant for the first time (OR = 0.56; 95% CI:0.34–0.96).

An increase in household income increased the odds of a woman receiving high quality ANC. Women from the highest income earning households were four times more likely to receive high quality ANC compared to women from lower earning households (OR = 4.35; 95% CI: 1.66–11.39).

### Factors associated with quality of antenatal care

In this study, quality of antenatal care is associated with several socio-economic and demographic factors. However, to assess the contribution of all these factors to the overall variance, multifactorial logistic regression analysis was used to control for confounding. Table 3 shows the final multifactorial model based on a backward investigator led logistic regression elimination method.

**Table 3:** Adjusted multifactorial logistic regression factors associated with high quality of antenatal care.

**Characteristics/factors**	**Study sample N (%)**	**Odds ratio (95% CI)**	***P*-value**
**HIGHEST LEVEL OF SCHOOL ATTENDED**			
Primary	189 (51.5)	1.0	
Secondary	133 (36.4)	1.98 (1.24–3.14)	0.004^*^
More than secondary (tertiary)	45 (12.3)	0.76 (0.35–1.65)	0.493
**CURRENTLY LIVING WITH PARTNER**			
No	226 (64.8)	1.0	
Yes	123 (35.2)	0.47 (0.28–0.79)	0.003^*^

* *p < 0.05*.

After adjusting for the effect of other factors, women with secondary education had higher odds of receiving high quality ANC than women with primary level of education (OR = 1.93; 95% CI: 1.17–3.15). Additionally, women currently staying with their husband/partners had lesser odds of receiving high quality ANC compared to those that were not staying with their husband/partner (OR = 0.47; 95% CI: 0.28–0.79).

## Discussion of results

This study examined the quality of ANC that pregnant women received during their most recent ANC visit. From the results, we were able to demonstrate that although first ANC attendance is high in Lusaka district, insufficient provision of certain key components of ANC was limiting the quality of antenatal care that women received. Among some ANC components not sufficiently provided was blood and urine testing, only 45 and 35% pregnant women received the mentioned test respectively. It is widely observed that testing services are insufficient both at country and district level. A national study conducted in 2012 found that only 60.8 and 22.6% of women provided blood and urine samples for testing, respectively (1).

Although first antenatal care visit is a success story in Zambia, only 47.1% of pregnant women received the recommended quality of ANC in Lusaka. This proportion is too low to achieve the full life saving potential that antenatal care could provide. Similarly, Nicholas found that only 29% of the women received high quality ANC in 2012 at national level (1).

Results of this study demonstrate that women with secondary education attainment were more likely to receive high quality ANC compared to those that attained primary level education. Equally, a study done in three African countries (Kenya, Malawi, and Nigeria) showed that women's education level has an effect on the quality of ANC. Educated women were more knowledgeable about the procedures to expect during ANC, hence more likely to request for such procedures than the low educated women (11). Another study conducted in south Asia suggested that education brings up new values and attitudes which upsurges the chances of a woman desiring skilled care and empowers them to access such care (12).

It is however, worth noting that there was no association between someone attaining higher education other than secondary level and the quality of antenatal care. A review of other studies by Mayura and Ikeoluwapo shows that the higher the educational attainment, the higher the odds of receiving high quality ANC (9, 13). Contrary to our findings, a study from southwest Nigeria on socio-demographic characteristics that affect pregnant women's perception of quality of ANC found that there was no association between level of education and quality of ANC. However, increasing parity, employment status and religion were found to have an influence on the quality of ANC. The study proposed intensive health center-specific key interventions on such women in order for them to receive quality ANC (14).

With relatively low literacy and education levels among the female folk in Zambia (68%) (3), quality of ANC is expected to be low. Therefore, there is need to supplement existing educational policies in order to improve female education which is likely to improve the quality of ANC.

Women's autonomy in decision making on health care has in recent years inadequately been researched in developing countries. This study explored the link between the quality of ANC between women who lived with their husband/partners and those that did not. There was a strong association between husband/partners presence and the quality of ANC a woman received. Studies have shown that women who lived with their partners were more likely to receive quality health care compared to those that were not staying with their partners (15).

A study done in Guinea Equatorial showed that most women who attended ANC were unmarried. Although most studies have shown how crucial spouses are toward influencing women to attend ANC (16), our study showed opposing results as it was observed that women staying with their husband/partners had lesser odds of receiving quality ANC.

Contrary to our findings, data from Nigeria showed that unsupported pregnant women (not staying with spouses) had lesser odds of utilizing available antenatal care services compared to supported women. The study further suggests that despite the marital status of the pregnant women, the presence of the spouse or partner was more likely to produce better results in insuring that women attend ANC (16). Despite a number of factors that influence women's health care decision making, our study showed that the presence of the husband/partner does not make it any better to receive high quality antenatal care compared to the absence of the partner.

Similar to our findings, a survey conducted in Southeast Asian Countries found that women staying with their partners had limited mobility to attend ANC due to partners control over attending or taking part in activities outside the house (17). This negatively affected the quality of ANC services that pregnant women received. Equally, our study showed that women staying with their partners had lesser odds of receiving high quality ANC.

## Limitations

The study sample obtained only consisted of women from government health facilities. This presents a missed opportunity to compare the quality of ANC between private and government facilities. Furthermore, this study only considered the process as well as outcome and overlooked the structure parameters in determining quality of ANC. Despite the stated limitations, the use of exit interviews for data collection as well as checking for correspondence of collected information on ANC cards enabled us to eliminate recall bias from our study hence increasing the quality of data collected.

## Conclusions

Our study examined the effect of different socio-economic and demographic factors that influence quality of ANC. After subjecting the data to all necessary statistical tests, level of education and husband/partners presence were found to be the major variables that affect quality of ANC. Women with secondary education had higher odds of receiving high quality ANC than women with primary education. On the other hand, pregnant women staying with their partners were less likely to receive high quality antenatal care compared to women not staying with their partners.

Despite an interplay of various factors that contribute to high maternal and neonatal mortality, the proportion of women receiving high quality ANC is too low to see the much needed reduction in mortality at district level. However, in the short term, continued efforts at improving delivery of basic services such as blood and urine testing is required.

We recommend that the Ministry of Health should develop tools to monitor and evaluate services provided from the patients' perspective. This will enable the District Medical Officers (DMOs) to supplement performance assessment results and also identify key areas that need capacity development and technical assistance (TA). The study further recommends that testing services be increased at facility level.

## Author contributions

BK, PB, CJ, and TH conception and design of the study. BK data analysis and drafting the article. BK and CJ interpretation of data. BK, PB, CJ, TH, and MC revising the article for critical intellectual content. PB, CJ, TH, and MC approval of the final version.

### Conflict of interest statement

The authors declare that the research was conducted in the absence of any commercial or financial relationships that could be construed as a potential conflict of interest.
